# Toward a unified theoretical model of bulimia nervosa: protocol for a systematic review and synthesis of existing theories

**DOI:** 10.1186/s13643-025-03047-z

**Published:** 2026-01-09

**Authors:** Kristine Schönhals, Vanessa Opladen, Lukasz Stasielowicz, Klaske A. Glashouwer, Silja Vocks

**Affiliations:** 1 Institute of Psychology, Department of Clinical Psychology and Psychotherapy, Osnabrück University, Osnabrück, Germany; 2 Department of Psychology, Division of Psychological Assessment, University of Salzburg, Salzburg, Austria; 3 Faculty of Behavioural and Social Sciences, Department of Clinical Psychology and Experimental Psychopathology, University of Groningen, Groningen, Netherlands; 4 Department of Eating Disorders, Accare Child and Adolescent Psychiatry, Groningen, Netherlands

**Keywords:** Bulimia nervosa, Eating disorders, Body image, Model, Theory

## Abstract

**Background:**

Bulimia nervosa is a severe mental disorder associated with several physical and mental health complications. The aim of this review is to provide an overview of existing theoretical models regarding the development and maintenance of bulimia nervosa. A second aim is to develop a synthesis to allow for a more complete understanding of bulimia nervosa, which may help to develop and refine interventions in the future.

**Methods:**

PsycInfo, PubMed, PSYNDEX, Scopus, and Google Scholar will be searched for studies presenting models of bulimia nervosa. Topic modeling will be used to gain initial insights into related factors. Additionally, two independent expert reviewers will screen the literature and select models of bulimia nervosa as well as associated datasets for additional empirical analyses. In the case of discrepancies, a third reviewer will be consulted. The similarities between the identified models of bulimia nervosa will be summarized in a narrative synthesis. Depending on the number of identified models and available datasets, models will be tested using existing datasets.

**Discussion:**

The review seeks to summarize existing models regarding the development and maintenance of BN. The results could be used as a starting point for developing adequate models, testing these models, and subsequently using them to inform evidence-based intervention programs in the future.

**Systematic review registration:**

This systematic review was registered with the International Prospective Register of Systematic Reviews (PROSPERO) on 30.09.2024 (CRD42024581179).

**Supplementary Information:**

The online version contains supplementary material available at 10.1186/s13643-025-03047-z.

## Background

### Rationale

According to the fifth edition of the Diagnostic and Statistical Manual of Mental Disorders [1], bulimia nervosa (BN) is an eating disorder that is mainly characterized by recurrent episodes of binge eating, associated with a perceived lack of control over eating, frequent compensatory behaviors such as vomiting, intake of laxatives, excessive sport, or dieting, as well as an overvaluation of shape and weight. The lifetime prevalence of BN is estimated at around 2–3% in females and more than 1% in males [2]. BN is a severe mental illness associated with an increased mortality rate [2, 3] and can lead to several medical complications [4]. Moreover, individuals with BN frequently suffer from psychological impairments such as sleep problems or suicidal thoughts, and often have comorbid mental disorders such as anxiety or mood disorders [5]. Overall, the efficacy of psychotherapy in terms of the remission of BN or the absence of BN symptoms after treatment is rather low, at around 40% [6, 7].

To explain the development and maintenance of BN, several models and theories have been proposed over the past decades. Some of these models cover eating disorders in general, e.g., the integrated cognitive-behavioral theory of eating disorders [8] or the tripartite influence model [9], while others were developed specifically for BN, e.g., the dual-pathway model [10]. The models vary substantially, e.g., in complexity, number of included factors, and proposed relationships between factors. For example, while the dual-pathway model [10] describes a much more narrowly defined area of BN pathology, in which body dissatisfaction is proposed as a central risk factor for BN, the integrated cognitive-behavioral model by Williamson et al. [8] encompasses several risk factors, situational cues, cognitive biases, behavioral variables, and feedback loops contributing to the development and maintenance of eating disorders in general.

A review by Pennesi and Wade [11] summarized various models of eating disorders that have informed the development of treatment programs. However, the review included only 10% of the models of eating disorders identified by the chosen search strategy. Thus, a great number of postulated key factors and relationships described in the remaining models were not explicitly considered in the review because the authors deemed them to be non-testable (e.g., models including more than six factors besides outcome variables). A broader consideration and evaluation of existing theoretical models might increase the strength of the evidence by identifying key aspects that are prevalent across different models and by ensuring that relevant aspects are not overlooked. Such an overview could help to explain and understand the pathogenesis and maintenance of BN and facilitate the development of adequate treatment programs. This synthesis may also contribute to advancing the field by improving the conceptualization of BN.

### Objectives

The intention of our systematic review is to identify relevant models of BN and to detect similarities and differences between the models concerning risk factors, onset factors, and maintenance factors of BN. First, relevant existing models will be searched for. Second, similarities between models will be examined. Third, models will be tested using the empirical data available in the literature. The data for testing the existing models will be extracted from studies that have explicitly examined existing models of BN or parts thereof and provide access to anonymized datasets. Based on the results, a synthesis will be proposed, which integrates key factors and relationships. The study protocol is based on the Preferred Reporting Items for Systematic Review and Meta-Analysis Protocols (PRISMA-P).

## Methods

### Eligibility criteria

Topic modeling (e.g., latent Dirichlet allocation) will be conducted with all abstracts yielded by the literature search in order to provide a preliminary overview of the literature. The theoretical models for the main analyses will be selected according to the following eligibility criteria: (1) Models that depict a conceptual or evidence-based model or theory regarding the development and maintenance of BN will be included. A model can depict either the development or the maintenance of BN or both. General models of eating disorders will be included if they also explicitly refer to BN insofar as they contain relevant information on the development and maintenance of BN. (2) Meta-analyses and reviews that cover relevant models will be included. (3) If the depicted model only refers to other eating disorders, such as anorexia nervosa or binge eating disorder, and does not refer to BN, the model will be excluded. (4) There are no limitations in terms of publication year. (5) Studies published in English, German, French, Polish, Swedish, Spanish, and Dutch will be included. There are no further eligibility criteria (e.g., regarding age, comorbidities, or gender), as we want our findings to be applicable to different BN backgrounds.

In addition, we will use accessible datasets of studies yielded by the search that aim to empirically test a model of BN or parts thereof. Furthermore, if the examined underlying model is not already included, we will search for the original study presenting the examined model.

### Information sources and search strategy

The five electronic databases PsycInfo, PubMed, PSYNDEX, Scopus, and Google Scholar will be searched. As we plan to include general models of eating disorders provided that they contain relevant information on the development and maintenance of BN, the search will not be restricted to BN. A broad search term will be used (e.g., not limited to titles or abstracts, including Medical Subject Headings (MeSH) and full texts, if possible) to enhance the likelihood of finding more relevant models. A filter will be applied to only search for models concerning humans. The search term will be used for two different purposes: (a) to find relevant models of BN and (b) to find datasets that have already been used to test these models or parts thereof.

An example of the search term for PubMed is (bulim* OR eating disorder) AND (model OR theor* OR framework). The term will be modified for each electronic database according to the specific guidelines.

### Study records

#### Data management

The metadata about the articles identified in the database search will be exported to EndNote. EndNote will be used to organize the articles, remove duplicates, and display titles and abstracts for screening.

#### Selection process

After removing duplicates, the article titles and abstracts will be screened by two independent reviewers for potential violations of the inclusion criteria. Following this screening, we will obtain full texts for the remaining studies that might include a relevant model or dataset. If the full text is not available, the corresponding authors will be contacted or the articles will be purchased. Two independent reviewers will then screen the full texts and decide on the inclusion of models in the review based on the described eligibility criteria. In the case of discrepancies between the two reviewers, a third reviewer will be consulted to resolve the disagreement. The selection process will be summarized in a PRISMA flow diagram. No reviewer will be blind to the journal titles, the study authors, or the institutions.

#### Data collection process and data items

Two raters will extract the following information from the eligible models independently from each other using a standardized codebook: (1) author, title, publication year, country of residence of the first author, (2) type of theory or model (e.g., global theory or model; depiction of only specific factors and links or mechanisms; restriction to certain settings such as social media use, etc.), (3) which eating disorders the model was developed for (only BN; models including other eating disorders besides BN), (4) factors depicted in the model (e.g., predictors, mediators and moderators, outcomes, maintaining factors). The data extracted by each rater will be compared, and potential discrepancies will be resolved by an independent third rater. If any uncertainties remain, the study authors will be contacted to resolve them.

#### Risk of bias

As we plan to use existing datasets that have already partially or fully examined a certain model, there is a potential risk of bias, as these datasets might favor the model for which they were originally used. Moreover, certain factors of some models might not be included in any of the available datasets. Furthermore, newer models and proposed factors might be underrepresented in the available datasets. To avoid overlooking these factors, the data synthesis will combine results from the data analysis and results from a narrative synthesis, and will take into account that more recent developments might be understudied.

### Data synthesis

To gain a first insight into factors that are often discussed in the context of BN, topic modeling (e.g., latent Dirichlet allocation) will be used. To this end, the content of all abstracts yielded by the search strategy will be analyzed, before screening individual articles and selecting models. This approach will allow us to identify common topics and keywords (e.g., risk factors, outcomes) in the extant literature.

Next, based on articles that introduce specific models of BN, we will conduct a narrative synthesis focusing on the similarities between the included models. Finally, if possible, existing datasets will be used to test postulated relationships and key factors described in the models in order to provide a preliminary assessment of the validity of the models. Depending on the number of identified models of BN and available datasets, several strategies can be used to empirically test model validity:

- Structural equation modeling can be used to directly test the relevance of variables (e.g., risk factors, mediators) and the relationships proposed by the models. To test models consisting of many variables, datasets with large samples are required.
- Meta-analytical structural equation modeling can be used to simultaneously test, across different samples, the relevance of variables (e.g., risk factors, mediators) and the relationships proposed by the models. This approach requires the availability of at least a few datasets containing information about the respective variables.
- Network analysis can be used to visualize relationships between certain items or scale scores (e.g., BN mean score, body dissatisfaction mean score) in individual datasets. This approach could help inform model modifications (e.g., additional paths connecting risk factors, mediators, and BN).

As it is unclear how many datasets and models will be found, it is not yet possible to formulate “cut-off criteria” for the inclusion of factors and relationships in a synthesis. One possible criterion could be the number of models that name a certain factor, with the cut-off being adjusted depending on the number of available models. Another criterion might be the strength of empirical evidence for proposed relationships, e.g., that certain relationships are statistically significant across different datasets and samples, or that the respective effect sizes are non-negligible. Crucially, it may not be possible to empirically test some complex models, and it is further important to consider that central variables of a particular model may not be covered in datasets that were originally used to test a different model of BN. Therefore, the information generated by the topic modeling, narrative synthesis, and the exploratory data analysis will be used together to successively propose a synthesis of existing models.

Potentially, more than one proposal for a unified theoretical model will emerge. In this case, the models will be tested on a preliminary basis using the existing datasets. In a next step, after this review, these proposals might be used by an expert panel to form a new model of BN.

## Discussion

The review seeks to summarize existing models regarding the development and maintenance of BN. Topic modeling will be conducted to provide a preliminary overview of the literature. After identifying relevant models, a narrative synthesis with a focus on similarities between the models will be conducted. If possible, models will be tested using existing data from studies identified through the literature search. The strength of this review lies in the combination of theoretical developments with empirical tests. Specifically, theoretical assumptions of various models of BN will be tested using existing datasets in order to provide more insight into the validity of these assumptions.

One limitation is that the review is based to a large extent on existing models, which are often heuristic in nature without sufficient empirical testing of proposed factors and relationships (e.g., Williamson et al. [8]). To mitigate this issue, we will conduct several analyses (e.g., topic modeling and network analysis) that can stimulate model modifications. Furthermore, available datasets will be used to examine several competing models. Nonetheless, the resulting proposals for a synthesis need to be tested in future longitudinal studies in order to verify the key factors and causal relationships proposed. The risk of bias inherent in using existing datasets to test models for which the data were not originally collected should be taken into account. The proposed synthesis should be carefully examined and could be used, for example, by an expert panel to make informed decisions about a BN model. This synthesis may contribute to advancing the field by improving the conceptualization of BN and highlighting the key directions for future research.

Notwithstanding the need to thoroughly examine the usefulness of the proposed synthesis in new samples, our multi-stage process of model refinement will ensure that the proposed synthesis provides a useful framework of key factors and relationships identified across different models in the extant literature. This framework will offer a more complete understanding of the development and maintenance of BN. As such, it could be used as a starting point for developing adequate models, testing these models, and subsequently using them to inform evidence-based intervention programs in the future.

## Supplementary Information

Supplementary Material 1: PRISMA-P checklist.

## Data Availability

The datasets used and analyzed during the current study are planned to be available from the corresponding author on reasonable request.

## Abbreviation

BN: Bulimia nervosa
